# Blood–brain barrier disruption: a pervasive driver and mechanistic link between traumatic brain injury and Alzheimer's disease

**DOI:** 10.1186/s40035-025-00478-5

**Published:** 2025-03-26

**Authors:** Bryan Sun, Lulin Li, Odette A. Harris, Jian Luo

**Affiliations:** 1 Palo Alto Veterans Institute for Research, VA Palo Alto Health Care System, Palo Alto, CA 94304 USA; 2 Department of Neurosurgery, Stanford University School of Medicine, Stanford, CA 94305 USA; 3 Polytrauma System of Care, VA Palo Alto Health Care System, Palo Alto, CA 94304 USA

**Keywords:** Blood–brain barrier, Traumatic brain injury, Alzheimer’s disease, Neuroinflammation, Neurodegeneration

## Abstract

Traumatic brain injury (TBI) has emerged as a significant risk factor for Alzheimer’s disease (AD), a complex and devastating neurodegenerative disorder characterized by progressive cognitive decline and memory loss. Both conditions share a common feature: blood‒brain barrier (BBB) dysfunction, which is believed to play a pivotal role in linking TBI to the development of AD. This review delves into the intricate relationship between TBI and AD, with a focus on BBB dysfunction and its critical role in disease mechanisms and therapeutic development. We first present recent evidence from epidemiological studies highlighting the increased incidence of AD among individuals with a history of TBI, as well as pathological and animal model studies that demonstrate how TBI can accelerate AD-like pathology. Next, we explore the mechanisms by which BBB dysfunction may mediate TBI-induced AD pathology. Finally, we investigate the shared molecular pathways associated with BBB dysfunction in both TBI and AD conditions and discuss the latest findings on how targeting these pathways and employing regenerative approaches, such as stem cell therapy and pharmacological interventions, can enhance BBB function and mitigate neurodegeneration.

## Background

The blood–brain barrier (BBB) is a selective permeability barrier that plays a critical role in maintaining the brain's homeostasis by regulating the passage of molecules and protecting the central nervous system (CNS) from harmful substances. The integrity of the BBB is essential for normal brain function, and dysfunction of this barrier has been implicated in a variety of neurological disorders, including traumatic brain injury (TBI) and Alzheimer's disease (AD). Both TBI and AD are characterized by BBB dysfunction, which can exacerbate neuroinflammation, disrupt neuronal function, and promote the accumulation and aggregation of neurotoxic proteins.

The aim of this review is to provide a comprehensive examination of the relationship between BBB dysfunction, TBI, and the increased risk of AD, with a particular focus on understanding the potential role of BBB disruption as a mechanism for AD development following TBI. A thorough search of the literature was conducted using major databases such as PubMed and Google Scholar, with key terms including "blood–brain barrier", "traumatic brain injury", "Alzheimer's disease", "BBB dysfunction", and "neuroinflammation". Studies published within the last 10 years were prioritized.

This review synthesizes the current understanding of how TBI-induced BBB dysfunction may act as a gateway to the development of AD. By examining the literature linking TBI to an increased risk of AD, the evidence of BBB dysfunction in both conditions, the molecular mechanisms underlying BBB disruption, and emerging therapeutic strategies, we aim to provide new insights into the potential of targeting BBB integrity as a means to reduce the risk of AD after TBI.

AD is one of the most intricate and devastating neurodegenerative disorders, impacting millions of people worldwide [1]. Although most late-onset AD (LOAD) cases are sporadic, various genetic and environmental risk factors have been identified that contribute to its onset and progression [2]. TBI is a well-established and potent risk factor for AD and has spurred extensive research to uncover the mechanisms linking these two debilitating conditions [3, 4]. Both conditions involve BBB dysfunction, which is believed to underpin their association. This review examines the complex relationship between TBI and AD, with a focus on BBB dysfunction and its crucial role in disease mechanisms and therapeutic development.

## TBI as a potent risk factor for AD

AD is characterized by cognitive decline, memory loss, and behavioral changes, with its pathology marked by the accumulation of amyloid-beta (Aβ) plaques and neurofibrillary tangles (NFTs) composed of hyperphosphorylated tau protein in the brain [2]. Vascular abnormalities, such as reduced cerebral blood flow and BBB disruption, are common in AD [5]. Aβ plaques, NFTs, neuronal loss, neuroinflammation, and cerebrovascular dysfunction are considered major contributors to AD pathophysiology and cognitive impairment [5, 6]. There is compelling evidence from epidemiological studies, pathological analysis, and animal research that TBI can trigger neurodegeneration, or increase the risk of neurodegenerative diseases like AD [3, 4].

### Epidemiologic studies

TBI as a risk factor for AD has been extensively studied in epidemiologic research. Although some findings are conflicting, the majority of studies, particularly large administrative and population-based cohort studies, demonstrate a positive association between TBI and an increased risk of developing AD [3, 4]. In a propensity score-matched study of over 350,000 veterans, dementia risk increases with TBI severity, with adjusted hazard ratios (HR) of 2.36 for mild TBI (mTBI) without loss of consciousness, 2.51 for mTBI with loss of consciousness, and 3.77 for moderate to severe TBI [7]. Similarly, a nationwide Danish cohort study of nearly 3 million people found an increased risk of all-cause dementia (HR 1.24) and AD (HR 1.16) in people with a history of TBI, with dementia risk increasing with the number of injuries (HR 1.22 for one TBI; HR 2.83 for five or more TBIs) [8]. Additionally, individuals with a history of TBI develop AD at a younger age [9]. Emerging evidence has led to the recognition of TBI as a potentially modifiable risk factor for dementia by the 2020 Lancet Commission on dementia prevention, intervention, and care [10].

Epilepsy is a common and severe consequence of TBI. Post-traumatic epilepsy (PTE) is a leading cause of acquired epilepsy, accounting for 5%–20% of such cases [11]. While epilepsy is independently linked to an increased risk of dementia [12], the dementia risk associated with PTE is significantly higher than that associated with TBI alone or non-traumatic epilepsy [13].

### Human pathological studies

The pathological link between TBI and AD involves TBI directly initiating or accelerating the neurodegenerative processes common to AD, such as neuroinflammation, oxidative stress, excitotoxicity, cerebrovascular impairment and BBB breakdown [14]. Moreover, TBI exacerbates pathological events specific to AD, including the accumulation of tau aggregates and Aβ plaques [15]. The shared neuropathology may explain the symptomatic overlap between TBI and AD and support TBI as a risk factor for AD. Upregulation of amyloid precursor protein (APP) and hyperphosphorylation of tau have been observed in human postmortem samples following TBI [16], and their heightened levels correlate with poor prognoses in TBI patients [17].

### Animal research

The pathological observations in TBI patients are largely recapitulated in experimental animal (wild-type) models of TBI [18], including repetitive mTBI [19], as well as moderate [20] and severe TBI [16]. Severe TBI not only induces self-propagating of widespread tau pathology but also facilitates the transmission of tau pathology between mice [16].

The TBI‒AD link is further strengthened by TBI exacerbating AD pathology and accelerating disease progression in AD models. Over the past 25 years, numerous studies have employed various injury systems in AD models, with recent studies focusing on mechanistic links between TBI and AD. For example, a recent study found that TBI promotes Aβ plaque and NFT formation by activating transcription factors like C/EBPβ, which upregulates delta-secretase (AEP) [21]. AEP cleaves APP and tau, driving Aβ production and tau hyperphosphorylation. Knocking out AEP or C/EBPβ reduces TBI-induced AD-like pathology and cognitive decline in the 3 × Tg AD mouse model [21]. AEP expression is also increased in TBI patients [21], highlighting the role of AEP in linking TBI to AD pathogenesis. Additionally, tau acetylation (ac-tau) has been identified as a key factor in TBI-induced tauopathy [22]. Blocking ac-tau reduces neurodegeneration and neurobehavioral impairment after TBI in wild-type mice [22] and in tauopathy models [23]. These findings link TBI to exacerbated tau pathology, reinforcing TBI as a risk factor for AD.

## BBB dysfunction: a prevalent pathology contributing to the pathogenesis of TBI and AD

The BBB is a highly specialized structure crucial for maintaining brain homeostasis and protecting it from potentially harmful substances [24]. Composed primarily of brain endothelial cells (BECs) interconnected by junction proteins, the BBB forms a selective barrier that regulates the passage of molecules into the brain. This selective permeability is further reinforced by pericytes, astrocytic endfeet, and the basal lamina, collectively called the neurovascular unit [24]. BBB permeability is a key indicator of its integrity, reflecting paracellular and transcellular transport [25]. Paracellular transport is regulated by tight and adherens junctions, which form seals between endothelial cells, while transcellular transport depends on specialized transporters managing molecule exchange in the brain [24]. Degeneration or shrinkage of BECs, coupled with the downregulation, degradation and mislocalization of tight junction proteins, disrupts paracellular transport. Similarly, impaired expression or function of BBB-associated receptors and transporters leads to dysregulated transcellular transport. BBB dysfunction may also involve other structures in the neurovascular unit, including pericyte degeneration or reduced coverage, basement membrane degradation, and swelling or detachment of astrocytic endfeet [25].

### BBB dysfunction after TBI

BBB dysfunction is a direct consequence of TBI [14] and often persists into the chronic stage [26], where it impacts acute pathology and long-term outcomes.

#### Evidence of BBB dysfunction after TBI

Clinical and animal studies show that BBB dysfunction is common and occurs rapidly after TBI [14, 27]. According to the two-hit (primary–secondary injury) hypothesis [27], primary BBB dysfunction results directly from the initial traumatic impact, causing microvascular shearing, hemorrhages, and dysregulated blood flow [28]. In contrast, secondary BBB dysfunction develops over time after the initial injury, driven by ongoing pathophysiological processes that exacerbate initial BBB damage, increase permeability, and compromise neurovascular integrity [27], seen in both acute and chronic stages [29].

Common vascular phenotypes reported from animal models of TBI include extravasation of plasma proteins, enhanced leukocyte adhesion to the vascular endothelium, downregulation of tight junction proteins, widening of intracellular junctions, acute pericyte loss, and disrupted communication between pericytes and endothelial cells (reviewed in [14, 30]). These results highlight the significant role of BBB dysfunction in TBI pathology [31–33] and its link to concussion susceptibility, with animals at higher risk showing increased BBB dysfunction [34].

#### Effects of BBB dysfunction on TBI pathology and long-term outcomes

TBI-induced BBB breakdown occurs rapidly after injury [35]. This disruption allows plasma proteins to enter the brain, triggering astrogliosis and impairing astrocyte function. These astrocytes lose expression of homeostatic proteins, which hinders BBB repair and neuronal recovery [36].

BBB dysfunction can persist long-term into the chronic stage following TBI [26], which is a potential driving force for TBI-associated chronic neurodegeneration [14, 31]. Pharmacologic restoration of the BBB 12 months after TBI remarkably prevents axonal neurodegeneration and promotes cognitive recovery [37]. This provides strong evidence that BBB dysfunction contributes to chronic neurodegeneration and that restoring BBB integrity, even in the chronic phase of TBI, can reverse neurodegeneration and improve cognitive function.

Chronic degeneration of mural cells and reduced expression of Caveolin-1 (cav-1) and LDL receptor-related protein 1 (LRP1) [38–40] may underlie the persistent BBB dysfunction. Cav-1 is critical for caveolae formation and transcytosis, while LRP1 facilitates the clearance of AD-related Aβ and tau at the BBB [33]. These changes compromise the BBB transit pathway for eliminating extracellular Aβ and tau, reducing their clearance from the cerebrovascular system.

BBB dysfunction has also been linked to PTE [41]. BBB dysfunction after TBI triggers an epileptogenic process through the transforming growth factor β (TGF-β)/albumin-mediated signaling pathway [27]. BBB breakdown allows serum albumin to accumulate in the brain, where it binds to TGF-β receptors on astrocytes, triggering astrogliosis and inflammatory responses [27]. This, in turn, further damages the BBB, creating a pathological feedback loop.

### BBB dysfunction in AD

BBB dysfunction is an early feature of AD, occurring before the onset of dementia or neurodegeneration and significantly impacting disease progression [33].

#### Evidence of BBB dysfunction in AD

BBB breakdown in AD has been confirmed through postmortem tissue analysis, neuroimaging, and biofluid biomarkers [33, 42]. Postmortem studies have revealed brain capillary leakage, degeneration of pericytes and endothelial cells, loss of tight junction proteins, brain infiltration of circulating leukocytes and red blood cells, and aberrant angiogenesis in AD [33, 42]. Neuroimaging has shown increased BBB permeability to gadolinium, microbleeds, diminished glucose transport and CNS leukocyte infiltration [33]. Biomarker studies indicate an increase in the cerebrospinal fluid (CSF)/serum ratio of albumin (Q_alb_) and IgG levels, with Q_alb_ serving as a potential prognostic biomarker [43]. In addition, recent advancements in RNA sequencing (RNA-Seq) have enabled detailed single-cell analysis of the BBB in AD. These studies reveal the presence of BBB dysfunction and its importance in AD, including the senescence and apoptosis of BECs, loss of pericytes, downregulation of P-glycoprotein (P-gp), vascular inflammation, and enrichment of AD risk genes in BECs [44–48].

Preclinical studies with rodent AD models provide insight into BBB impairment in relation to disease progression. They show that BBB dysfunction occurs early, before Aβ pathology and cognitive decline [32]. Importantly, restoring BBB integrity reduces AD pathology [49]. However, not all findings support the notion that BBB breakdown is an early and central feature of AD [50]. It remains unclear whether these results reflect limitations of the animal models or suggest a lack of involvement of BBB dysfunction in AD.

#### Effects of BBB dysfunction on AD pathology

BBB dysfunction may directly impair Aβ clearance, leading to increased Aβ accumulation in the brain [33]. Under physiological conditions in the mouse brain, the BBB clears 80%–85% of AD-related Aβ through transvascular transport, a process that requires P-gp and LRP1. In AD, both transporters are downregulated, leading to reduced Aβ elimination and increased Aβ accumulation within the brain parenchyma [33]. Indeed, inhibition of P-gp [51] or LRP1 [52] worsens Aβ pathology and cognitive impairments in AD mice.

BBB dysfunction in AD can also contribute to pathology and neurodegeneration through neuroinflammation. Increased BBB permeability allows blood-derived neurotoxic substances, immune cells, and inflammatory mediators to infiltrate the brain, activating microglia and astrocytes, which accelerates neuronal damage and cognitive decline [33]. Fibrinogen, a plasma protein that accumulates rapidly in both AD mouse and human AD brains [53], is associated with neurodegeneration and cognitive dysfunction [54]. Targeting fibrinogen with a monoclonal antibody suppresses neurodegeneration and inflammation in the 5 × FAD mouse model of AD [55].

#### Contribution of Aβ and tau to BBB dysfunction in AD

While BBB dysfunction aggravates Aβ deposition and tau phosphorylation, the deposition of Aβ and tau can further damage BBB [56]. Chronic exposure to Aβ leads to oxidative stress and apoptosis in BECs, with matrix metalloproteinase (MMP) activation degrading tight junction proteins, further impairing BBB integrity [57]. Aβ aggregates, which vary in size and structure, have different toxic effects on the cerebrovasculature. In particular, Aβ fibrillar deposits inhibit angiogenesis and increase BBB permeability [58], linking Aβ aggregation to BBB dysfunction.

BBB damage can occur in tauopathies without Aβ overproduction and is suppressed by reducing tau expression, suggesting that tau alone is sufficient to cause BBB dysfunction [59]. BBB breakdown in tauopathies is thought to be driven by glial activation [60]. However, in P301S transgenic mice, soluble tau aggregates are internalized by BECs, resulting in mitochondrial damage and BEC senescence [61], suggesting direct toxicity of tau to BECs. Therefore, BBB damage in tauopathies likely involves multiple mechanisms.

**Fig. 1:**
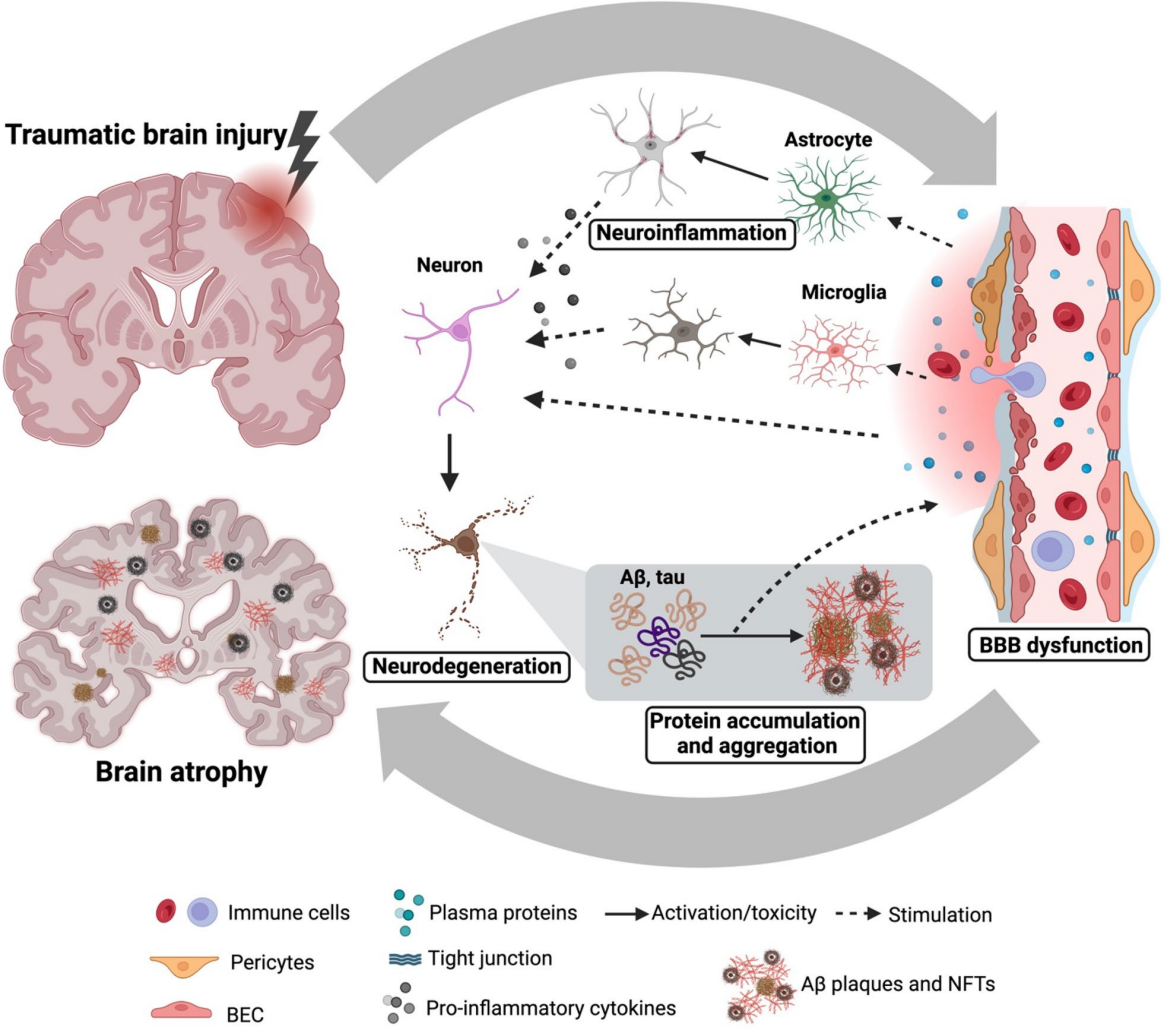
Blood‒brain barrier (BBB) dysfunction at the center of pathologic events in mTBI leads to neurodegeneration and AD. Upon impact, the primary brain injury damages the brain vasculature. Vascular dysfunction and BBB leakage not only impair the elimination of neurotoxic Aβ and tau proteins through the vasculature but also allow blood-borne proteins and immune cells to enter the brain parenchyma, exacerbating reactive gliosis and perpetuating neuroinflammation. Reactive astrocytes and microglia release proinflammatory cytokines, which, along with plasma proteins, promote the accumulation and aggregation of Aβ and tau. Ultimately, this cascade results in neurodegeneration and brain atrophy. The accumulation of Aβ and tau can, in turn, damage the BBB. Created with BioRender.com

In summary, BBB dysfunction is an early and common event after TBI and in AD. Current evidence suggests that BBB dysfunction may drive disease progression and contribute to the chronic neurodegenerative process of both TBI and AD, supporting a potential mechanistic link between the two [31] (Fig. 1).

## Common molecular mechanisms of BBB dysfunction in TBI and AD

Although the initial events causing BBB dysfunction in TBI and AD differ, existing evidence indicates an overlap in the molecular mechanisms underlying BBB pathology under both conditions. This overlap provides a mechanistic link between TBI and AD at the molecular level, presenting opportunities to develop potential therapeutic targets.

### Apolipoprotein E4 (APOE4)

Human APOE has three isoforms: APOE2, APOE3, and APOE4. In the brain, APOE is mainly produced by astrocytes and neurons, with astrocytes accounting for approximately 75%–80% of the total APOE in the mouse brain [62]. After production, around 60% of astrocytic APOE is secreted into the extracellular space, while the majority of APOE produced in neurons stays intracellular [62]. Other cell types, such as microglia, oligodendrocytes, and vascular cells have been shown to produce APOE at different levels, but their contributions to the total and the secreted brain APOE are not yet defined [62]. *APOE4* is the strongest genetic risk factor for early- and late-onset AD [62] and the best-known genetic risk factor for poor outcomes after TBI [63]. Studies from AD patients, patient-derived stem cell models, and human *APOE4*-expressing and *APOE*-deficient mouse models clearly demonstrated that APOE4 provokes neuroinflammation, impairs the cerebrovasculature, and exacerbates Aβ and tau pathology [64].

**Fig. 2:**
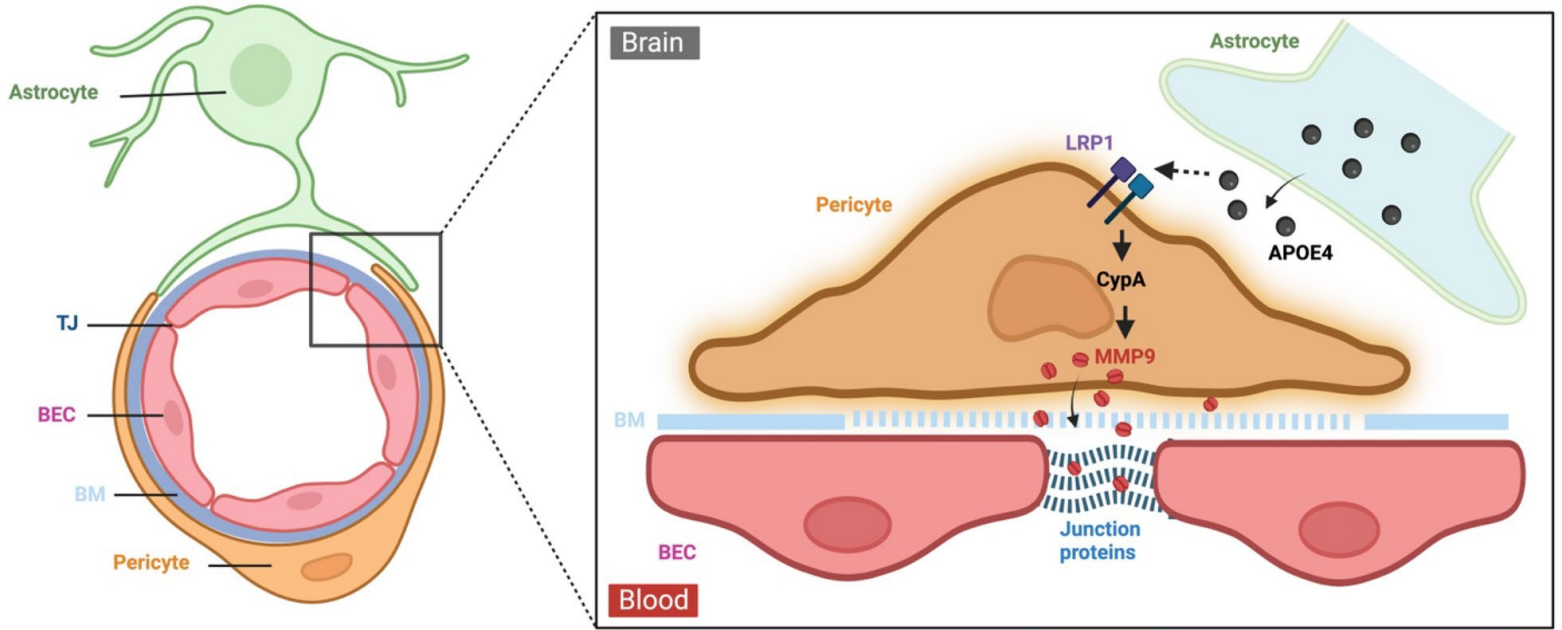
Regulation of BBB permeability by APOE4. APOE4 secreted by astrocytes has a low binding affinity for the low-density lipoprotein receptor-related protein 1 (LRP1) on pericytes. This weak binding activates the proinflammatory CypA/MMP-9 pathway, which results in release of MMP-9 into the extracellular space. Here, MMP-9 enzymatically degrades the basement membrane (BM) and junction proteins, leading to BBB breakdown. Created with BioRender.com

APOE4 affects the functions of nearly every cell type in the brain. Its regulation of BBB function is a key mechanism underlying its effects on neurological outcomes [62]. Transgenic expression of *APOE4* in astrocytes, but not *APOE2* and *APOE3*, leads to BBB breakdown in mice lacking murine ApoE [65]. BBB impairment is also observed in aged *APOE4* knock-in mice [66]. BBB dysfunction in these mice is further demonstrated by early disruption of the BBB transcriptome [67]. Individuals carrying *APOE4* exhibit increased BBB permeability [68] and distinct transcriptional signatures indicative of BBB dysfunction in BECs and pericytes [45–47]. In addition to increasing BBB permeability, APOE4 also impairs BBB-mediated Aβ clearance [62]. The changes caused by APOE4 at the BBB, which is considered to contribute to cognitive decline [69, 70], are further exacerbated by TBI [71] or AD [70]. The effects of APOE4 on BBB permeability are linked to the activity of MMP-9, which enhances paracellular permeability [65]. The activation of MMP-9 pathway has been demonstrated in *APOE4* transgenic mice [65], in *APOE4* knock-in mice following TBI [71], in *APOE4* knock-in mice crossed with 5 × FAD mice [70], as well as in AD patients with the *APOE4* genotype [72]. Consequently, worse clinical outcomes following repetitive mTBI [73] and increased prevalence and severity of CTE [74] are observed in *APOE4* carriers. Although APOE4 has consistently been shown to activate the MMP-9 pathway, the underlying mechanism remains unclear. It has been shown that unlike APOE3 that binds LRP1 to inhibit NF-κB-dependent MMP9 activation, thus conferring protection of the BBB, APOE4 has a low binding affinity to LRP1, leading to the activation of the CypA/MMP-9 pathway in pericytes [65] (Fig. 2). However, other studies indicate that APOE4 binds LRP1 with the highest affinity among the APOE isoforms [75]. This suggests that APOE4 may activate MMP-9 through an LRP1-independent pathway [66].

Besides APOE4 expression in astrocytes [65, 66], expression of APOE4 in vascular mural cells [76], border-associated macrophages (myeloid cells in close proximity to neocortical microvessels) [77] or in the periphery [78] results in neurovascular dysfunction. However, the underlying molecular mechanisms remain unknown.

The detrimental effects of APOE4 in diverse disease contexts provide a strong rationale for reducing APOE4 as a therapeutic approach [64, 79]. This finding is supported by numerous observations that genetic knockdown or depletion of APOE4 in mouse models strongly attenuates neuroinflammation and alleviates Aβ and tau pathology [64]. Several therapeutic strategies have been developed, including blocking APOE interactions, decreasing APOE4 levels, and increasing APOE lipidation [80]. Among these, APOE antisense oligonucleotides [81], APOE siRNAs [82] and APOE antibodies [83] have been shown to protect against Aβ and tau pathology as well as associated neuroinflammation and neurodegeneration. Excitingly, APOE immunotherapy with an anti-human APOE antibody also rescues cerebrovascular dysfunction [83].

### TGF-β

**Fig. 3:**
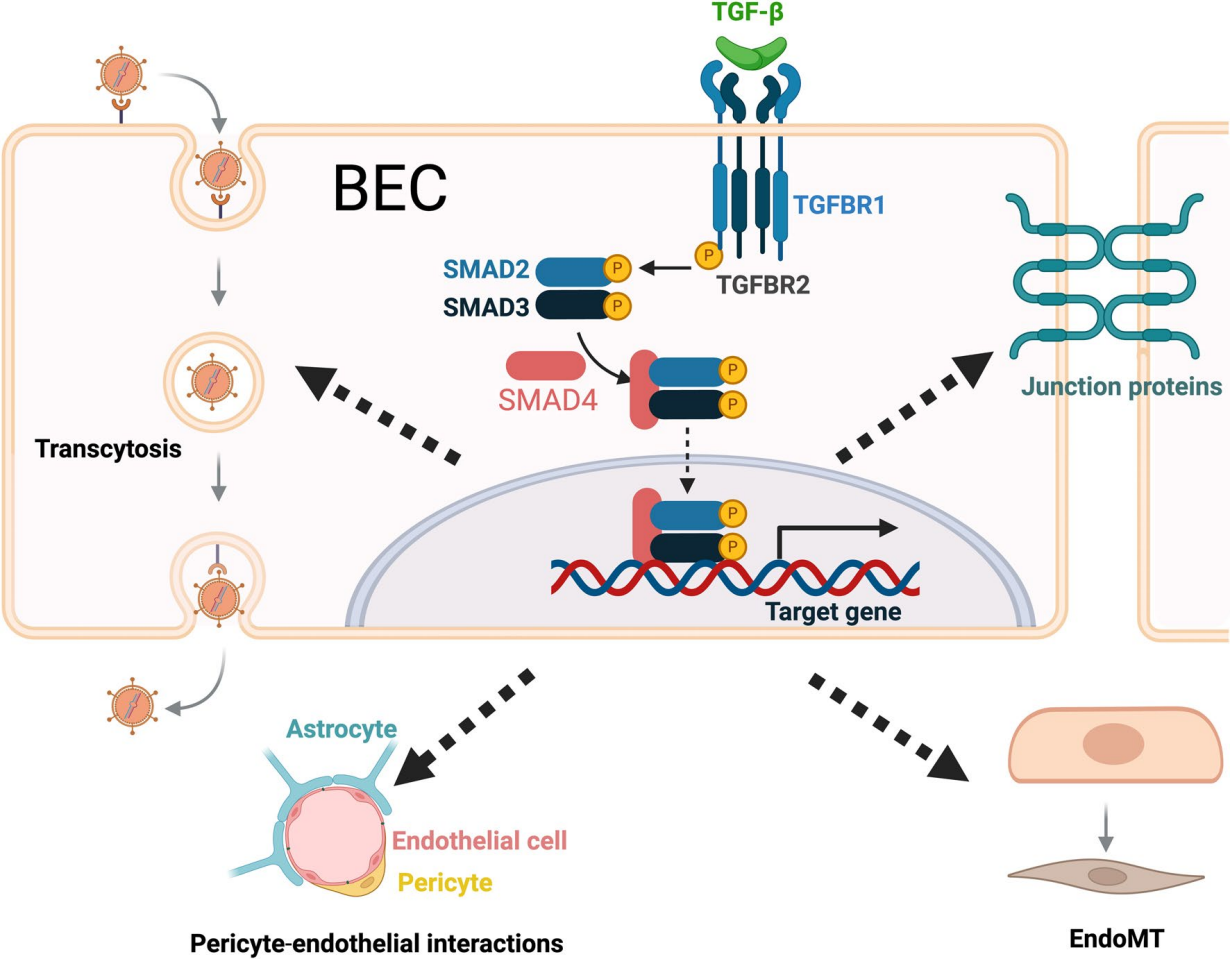
Multifaceted roles of TGF-β signaling in BBB integrity. In the canonical pathway, TGF-β binding to the TGFBR1 and TGFBR2 receptors leads to the phosphorylation and activation of TGFBR1. Activated TGFBR1 then phosphorylates SMAD2 and SMAD3. These phosphorylated SMADs form a complex with SMAD4 and translocate to the nucleus, where they regulate gene expression. Activation of TGF-β signaling can regulate multiple aspects of BBB Integrity: impacting transcytosis, expression of junction proteins, interactions between endothelial cells and pericytes, and endothelial-to-mesenchymal transition (EndoMT). The effects of TGF-β signaling activation are highly context-dependent. Dysregulated TGF-β signaling after TBI and in AD impairs BBB integrity. Created with BioRender.com

The TGF-β superfamily comprises a diverse group of pleiotropic cytokines with essential roles in various biological processes. In the CNS, TGF-β and its receptors are widely expressed across various cell types, where they regulate a wide range of microglial, astroglial, neuronal, and endothelial functions, playing an essential role in multiple aspects of brain function [84–86]. Dysregulated TGF-β signaling is implicated in the pathogenesis of neurological disorders [86]. In the vasculature, TGF-β has been identified as a key regulator of vascular development and angiogenesis [87]. Perturbed TGF-β signaling contributes to vascular pathologies [84]. Under healthy conditions, TGF-β promotes the expression of tight junction proteins [88], supports the interactions between endothelial cells and pericytes, mediates the recruitment of astrocytes to the BBB, and modulates astrocyte behavior [84] (Fig. 3). Disruption of the TGF-β signaling pathway typically leads to abnormalities in the vasculature and BBB integrity. For example, depletion of Smad4, a central intracellular mediator of TGF-β signaling, specifically in BECs, results in perinatal intracranial hemorrhage and BBB breakdown [89].

In neurological diseases such as AD and TBI, the expression of TGF-β and its signaling components in the neurovasculature is altered, leading to dysregulated TGF-β signaling and subsequent BBB dysfunction [84]. The effects of TGF-β signaling on the BBB are complex and depending on disease and stage, can have both beneficial (such as maintaining BBB integrity) and detrimental roles (such as promoting BBB breakdown). TGF-β signaling influences BBB integrity through multiple mechanisms, including alteration of tight junction protein expression [90], modulation of endothelial cell function and endothelial-to-mesenchymal transition [91], and induction of inflammatory responses that disrupt the vascular permeability [34]. In TBI, controversial findings have been reported regarding the expression of TGF-β and the components of the pathway, as well as the effects of modulation of TGF-β signaling [86]. In AD, however, the findings are more consistent, with TGF-β signaling generally being reduced [86]. This reduction is associated with decreased expression of tight junction proteins and disruption of interactions between endothelial cells and other components of the BBB [84]. In support of the critical role of TGF-β in the BBB in AD, multiple independent single-cell/nuclear RNA sequencing studies [44–47] have recently identified TGF-β signaling as a major regulator of AD pathology in vascular cells. More specifically, a recent single-nucleus RNA sequencing study revealed that dysregulated pericytic SMAD3 (a main downstream effector of TGF-β) signaling is responsible for BBB dysfunction in AD [92].

The effects of TGF-β signaling are complex and highly context- and cell type-dependent [86]. Similarly, AD is a multifactorial disorder characterized by a wide range of cellular and molecular abnormalities affecting multiple brain cell types [6]. These complexities highlight the need for a holistic treatment approach that targets not only specific cellular processes but also the interactions among diverse cell types and systemic factors.

### Major facilitator superfamily domain containing 2A (Mfsd2a)

**Fig. 4:**
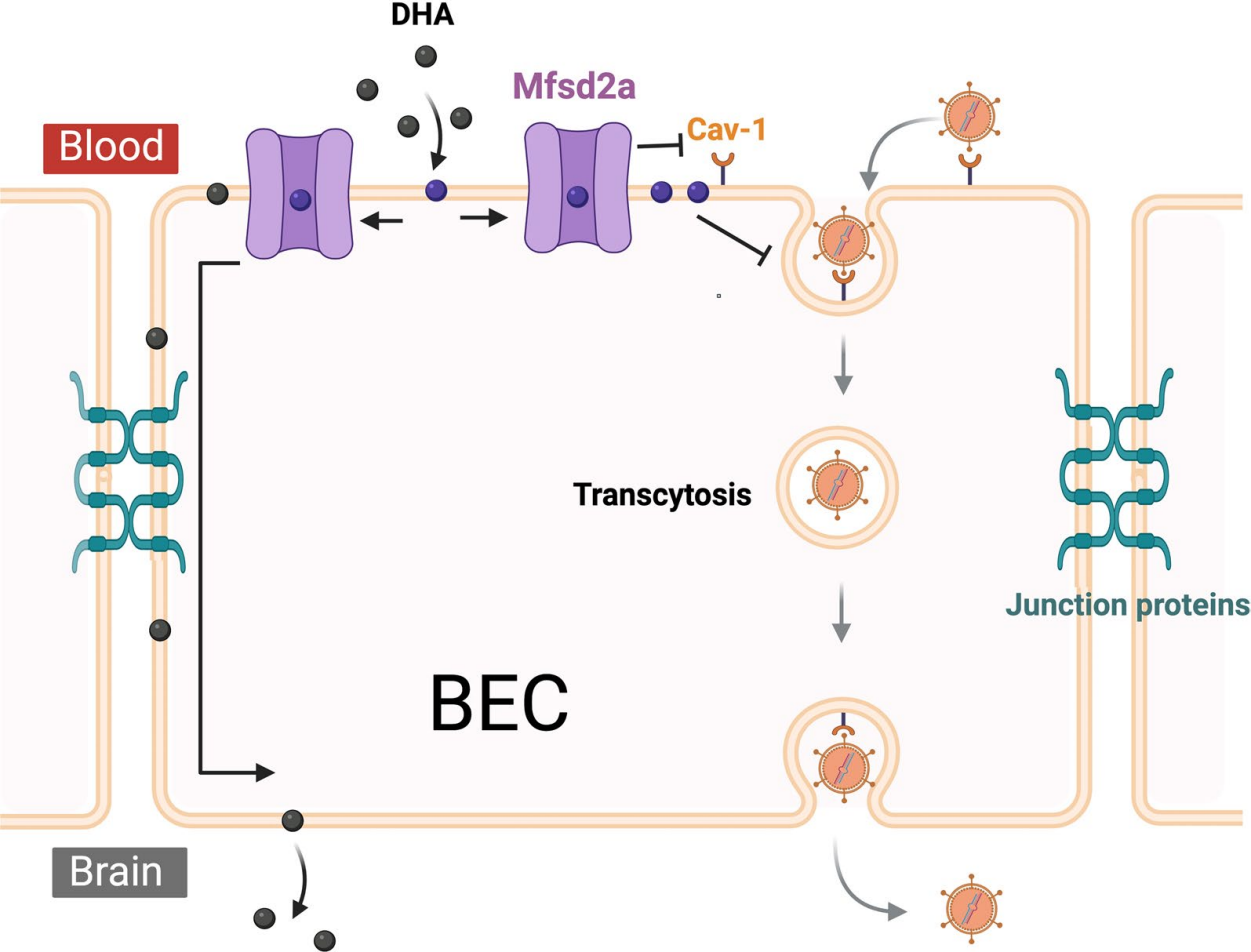
Regulation of BBB permeability by Mfsd2a. Mfsd2a is expressed on the luminal surface of BECs at the BBB, where it facilitates LPC-DHA uptake from plasma. This transport regulates the plasma membrane composition of BECs, inhibiting Cav-1-positive caveolae vesicle formation and suppressing the caveolae transcytosis pathway. Overexpression of Mfsd2a further downregulates Cav-1 expression, amplifying the inhibition of transcytosis. However, following TBI or during AD, Mfsd2a expression is reduced, resulting in increased caveolae vesicle formation and enhanced transcytosis across endothelial cells. Created with BioRender.com

Mfsd2a is a sodium-dependent lysophosphatidylcholine (LPC) transporter essential for the regulation of the BBB and the uptake of omega-3 fatty acids, particularly docosahexaenoic acid (DHA), into the brain. Mfsd2a is selectively expressed in BECs and is essential for the proper formation and maintenance of BBB integrity, acting as a key inhibitor of transcytosis [93]. The active suppression of transcytosis is critical for maintaining BBB function [94]. Genetic deletion of Mfsd2a leads to BBB defects from embryonic day 15.5 to adulthood due to increased transcytosis across the endothelium [93]. The suppression of the transcytosis pathway depends on Mfsd2a-mediated transport of lipids, particularly DHA-containing phospholipids, which regulate the plasma membrane composition of BECs and inhibit caveolae vesicle formation [95] (Fig. 4).

In TBI, Mfsd2a expression is reduced [96–98]. Restoring Mfsd2a expression has been associated with improved BBB function, lessened brain edema and abolished neurologic impairment in TBI models [96], suggesting its potential as a therapeutic target for enhancing brain repair and functional recovery postinjury. The beneficial effect of Mfsd2a overexpression on BBB function is associated with diminished expression of Cav-1 [96–98]. In AD, Mfsd2a levels in the blood significantly and progressively decrease, making it a potential biomarker for this disease [99]. A comparison of Mfsd2a mRNA and protein expression in 4- and 12-month-old 5 × FAD and WT mice revealed an age-dependent downregulation of Mfsd2a, which was exacerbated in 5 × FAD mice [100]. 5 × FAD mice crossed with *APOE4* knock-in mice exhibit further Mfsd2a downregulation; human *APOE4* carriers show a similar reduction [101]. Therefore, it is likely that the *APOE* genotype influences Mfsd2a function. Enhancing Mfsd2a expression or function could therefore serve as a potential therapeutic approach to restore BBB integrity, especially in *APOE4* carriers who are at an increased risk for AD. However, therapeutic approaches aimed at increasing Mfsd2a function or mimicking its activity, particularly concerning BBB integrity, have not yet been tested directly in AD models. Notably, virally induced overexpression of Mfsd2a has been shown to improve cognitive function due to chronic cerebral hypoperfusion [102], a condition that strongly exacerbates Aβ accumulation [103]. Therefore, similar therapeutic effects could be achieved in AD.

### Wnt/β-catenin signaling

The Wnt/β-catenin signaling pathway is a key regulator of cell proliferation, migration, and differentiation. In the brain, this pathway is essential for neuronal survival and neurogenesis, playing critical roles in regulating synaptic plasticity as well as the integrity and function of the BBB [104, 105]. Wnt7a and Wnt7b, ligands primarily produced by neurons and astrocytes in the brain, activate Wnt/β-catenin signaling in BECs [106]. This signaling pathway regulates the expression of tight junction proteins, influence vascular formation, and promote endothelial cell differentiation, thereby preserving BBB integrity [107]. Adult mice with endothelial-specific β-catenin knockout exhibit widespread leakage of plasma IgG and albumin, reduced tight junction protein expression, downregulated Mfsd2a, and increased caveolae-mediated transcytosis [108]. Abnormal Wnt signaling also disrupts interactions between oligodendrocytes and the vasculature, leading to BBB damage and subsequent CNS inflammation [109]. Wnt signaling is impaired in AD [110]. Exposure to Aβ and APOE4 inhibits the canonical Wnt/β-catenin pathway [111, 112].

**Fig. 5:**
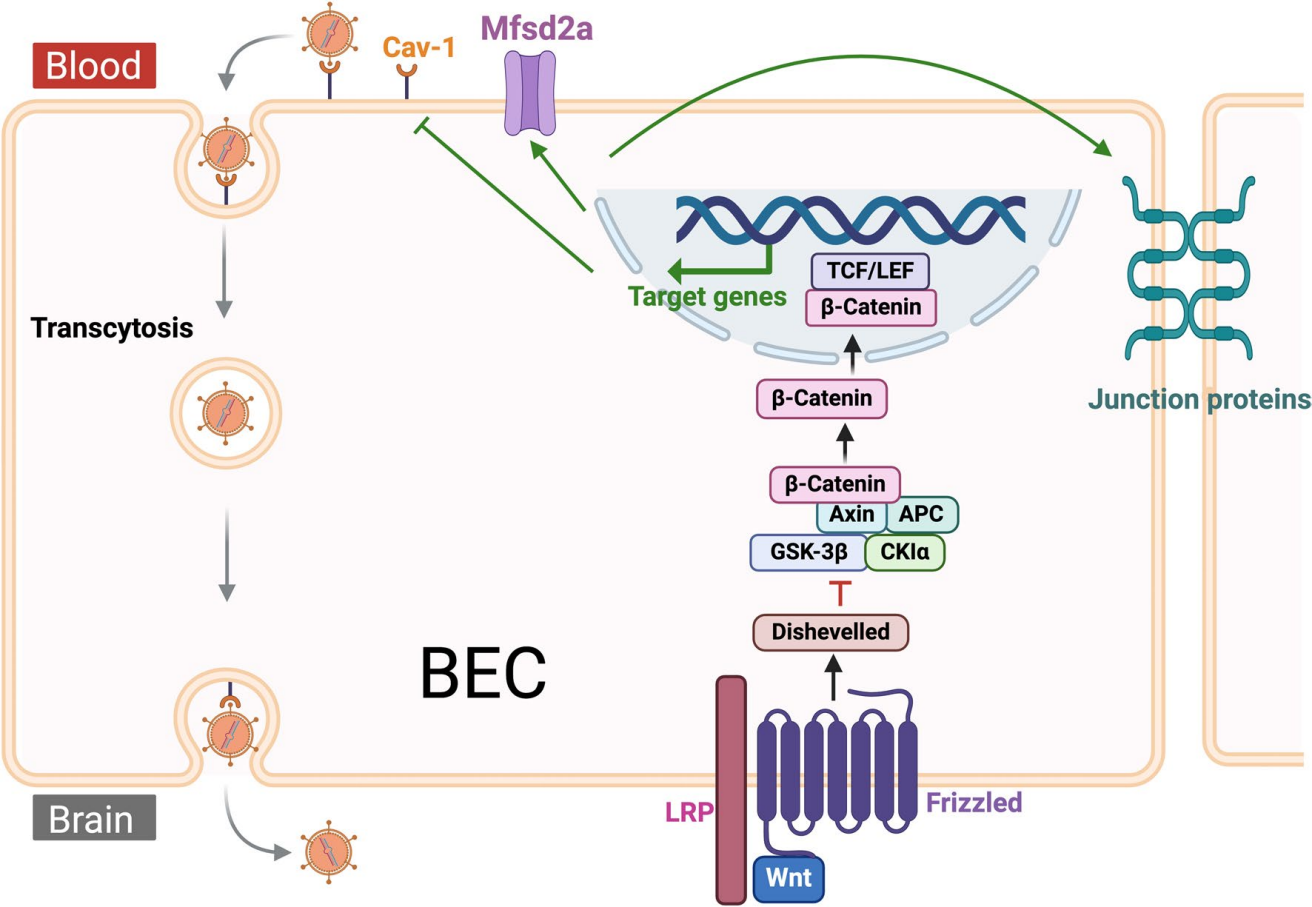
Activation of the Wnt/β-catenin signaling pathway maintains the BBB integrity. The presence of Wnt ligand or β-catenin activates the Wnt/β-catenin signaling pathway, regulating target gene expression. This activation upregulates Mfsd2a and downregulates Cav-1, thereby reducing endothelial transcytosis. It also increases the expression of junction proteins, strengthening tight junctions. Together, the reduction in transcytosis and the enhancement of tight junctions collectively maintain BBB integrity. In the disease state, the Wnt/β-catenin signaling pathway is compromised, resulting in weakened tight junctions, increased endothelial transcytosis, and subsequent BBB breakdown. Created with BioRender.com

Extensive research has explored the ability of activating the Wnt/β-catenin pathway to protect BBB integrity and mitigate neurodegenerative diseases. Recent advancements include the engineering of Wnt7a ligands specifically for BBB activation [113] and the use of FZD4 surrogate treatment [114]. Mechanistically, the therapeutic effects of activating endothelial Wnt signaling on BBB integrity involve restoring the expression of functional proteins, such as the tight junction protein Claudin-5 [115] and the transcytosis-inhibiting Mfsd2a [98, 113, 116] (Fig. 5). In TBI, the upregulation of Wnt/β-catenin signaling is associated with vascular repair [117]. The intranasal application of recombinant Wnt3a promotes BBB repair and recovery after TBI [118]. Pharmacological activation of Wnt signaling mitigated BBB disruption and improved neurological outcomes after TBI in mice [98]. Similarly, in the APP/PS1 mouse model of AD (APPswe/PSdE9 double transgenic mice), activation of the Wnt/β-catenin pathway via optogenetic stimulation of LRP6 (an upstream regulator of Wnt signaling) restored BBB function and ameliorated AD pathology [119]. The dysregulation of Wnt signaling in AD is further evidenced by the disruption of its downstream pathways. Death receptor 6 (DR6), a downstream target of the Wnt/β-catenin pathway, is highly expressed in the brain vasculature but significantly reduced in BECs of the APP/PS1 murine model of AD. Overexpression of DR6 is sufficient to rescue BBB dysfunction in vitro [120]. These studies underscore the importance of the Wnt/β-catenin signaling pathway in BBB dysfunction in both TBI and AD.

### MMP-9

MMPs constitute a family of zinc-dependent endopeptidases crucial for regulating numerous physiological processes, such as activating growth factors, cleaving zymogens, and remodeling the extracellular matrix [121]. They are initially synthesized as inactive zymogens and are activated through proteolytic removal via cysteine‒zinc interactions. Among the MMP family members, MMP-9 is the most abundantly expressed and extensively studied in the CNS because of its impact on BBB integrity. MMP-9 can be activated by oxidative stress, proinflammatory cytokines, chemokines, and infiltrating or resident inflammatory cells, all of which are common in TBI and AD pathology. Neutrophils are a major source of MMP-9, but it is also expressed by other infiltrating leukocytes, endothelial cells, and, to a lesser extent, astrocytes and neurons [122] (Fig. 7). Notably, pericytes can serve as a primary source of MMP-9 under specific inflammatory conditions [65]. The activation of MMP-9 leads to the degradation of tight junction proteins, which are crucial for maintaining the integrity of the BBB [123]. MMP-9 also targets components of the basement membrane surrounding cerebral blood vessels, such as collagen IV, laminin, and fibronectin, further compromising BBB function [123] (Fig. 2). MMP-9 binds to and cleaves lipoprotein receptors, triggering ectodomain shedding. The proteolytic cleavage of LRP1 and low-density lipoprotein receptor (LDLR) by MMP-9 disrupts their capacity to transport Aβ at the BBB [124]. In TBI, MMP-9 expression is significantly increased in neutrophils following initial injury and plays an important role in the recruitment of neutrophils into the damaged brain parenchyma [125]. Compared with wild-type mice, MMP-9 gene knockout mice show decreased BBB permeability, lessened fibrinogen deposition and improved memory following TBI [126]. Therefore, MMP-9-mediated BBB dysfunction is considered a major pathway leading to the development of vasogenic cerebral edema, elevated intracranial pressure (ICP), poor cerebral perfusion, and brain herniation following TBI [123]. In AD, MMP-9 can be activated directly by the presence of Aβ [127]. Treatment with an MMP-9 inhibitor or deletion of the MMP-9 gene in 5 × FAD mice improved sociability and social recognition memory [128].

MMP-9 expression and activity are modulated by ApoE4, as elevated levels of MMP-9 are observed in the cerebrovasculature of both human and animal AD brains with an *APOE4* genotype [66, 72] (Fig. 2). MMP-9 mediates *APOE4*-driven BBB dysfunction after TBI [71]. These findings indicate that MMP-9 plays a critical role in mediating the BBB dysfunction associated with the *APOE4* genotype, highlighting a potential mechanistic link between MMP-9 activity, ApoE4, and BBB dysfunction in both TBI and AD.

#### Sonic hedgehog (Shh) signaling

Shh signaling is a pivotal pathway in neural development and adult brain function and significantly influences TBI and AD [129]. In the context of the BBB, Shh signaling contributes to the development and maintenance of the BBB by stimulating tight junction proteins and endothelial cell function [130]. In neurological conditions such as TBI, Shh signaling has been implicated in neuroprotection and repair processes. The expression of endogenous Shh and Gli-1 (a downstream signaling molecule of the Shh pathway) is downregulated after TBI, suggesting impaired Shh signaling during the acute phase. Boosting Shh signaling through exogenous Shh treatment attenuates neuron death and BBB dysfunction while promoting neurological recovery after TBI [131]. In addition, the activation of Shh signaling has been shown to reduce TBI-induced seizures [132] while stimulating perilesion cell proliferation and improving function after TBI [133]. The downregulation of Shh after TBI appears to be mediated by endothelin-1 (ET-1) through activation of the endothelin ET_B_ receptors. ET_B_ receptor antagonism promotes Shh production and reduces BBB leakage after TBI. Therefore, endothelin-induced downregulation of Shh likely promotes BBB disruption [134]. In AD, Aβ disrupts Shh signaling [135]. This pathway regulates neurogenesis and neuronal survival, and inhibiting it has been shown to lead to neurodegeneration [129], suggesting a protective role of Shh signaling against AD-related neurodegeneration. On the other hand, a detrimental role of Shh signaling has also been reported. Shh is involved in the upregulation of APP and Aβ during aging [129]. Blocking the Shh pathway via overexpression of the protease nexin-1 reduces neuronal apoptosis and improves spatial learning and memory in AD mice [136].

**Fig. 6:**
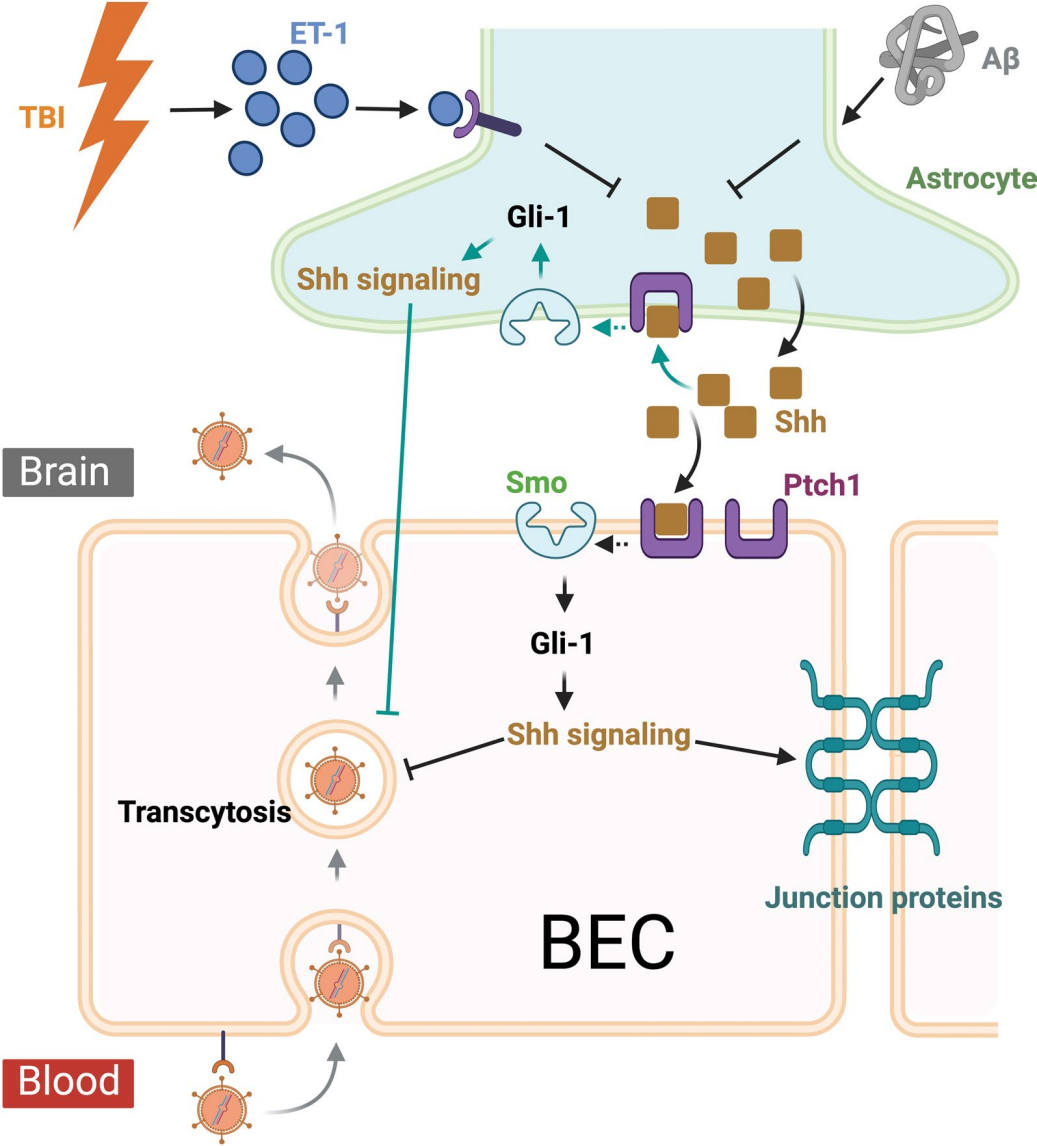
Potential mechanisms of Shh signaling pathway in maintaining BBB integrity. Model 1 (Shown by black lines and arrows): Astrocytes secrete Shh, which binds to its receptor Ptch1 on BECs to activate Smo and initiate the transcription of Gli1 and other target genes. This signaling leads to the upregulation of junctional proteins that restrict paracellular diffusion across the BBB, along with a reduction in transcellular transport. Model 2 (Shown by green lines and arrows): Binding of Shh to the Ptch1 receptor on astrocytes activates downstream Shh signaling targets, including Gli1, leading to reduced transcytosis in BECs. After TBI or in AD, Shh expression is suppressed, leading to increased endothelial transcytosis and BBB permeability. The downregulation of Shh following TBI is mediated by Endothelin-1 (ET-1), which activates endothelin ET_B_ receptors. Created with BioRender.com

The role of Shh signaling in maintaining BBB integrity was initially identified through astrocytes [130]. Specifically, astrocytes secrete Shh, which binds to its receptor Ptch1 on BECs to activate Smo and initiate the transcription of Gli1 and other target genes. This signaling leads to the upregulation of junctional proteins that restrict paracellular diffusion across the BBB. Additionally, Shh signaling in BECs increases transendothelial electrical resistance and reduces transcellular transport [130] (Fig. 6). These results indicate that activation of Shh signaling in BECs promotes BBB integrity through both transcellular and paracellular mechanisms. However, recent transcriptional profiling studies have revealed that the core Shh-pathway components, including Ptch1, Smo, and Gli genes, are specifically expressed in protoplasmic astrocytes and not in BECs [137]. Importantly, conditional inactivation of Shh signaling specifically in astrocytes results in a transient, region-specific BBB leakage, by increasing transcytosis without affecting paracellular diffusion [137] (Fig. 6). While these findings support the crucial function of Shh in maintaining BBB integrity, they demonstrate that astrocytes, rather than BECS, are the primary Shh-responsive cell population in the CNS. While the reason for this difference is unclear, it is consistent that Shh signaling deficiency, whether in astrocytes or in BECs, leads to impaired BBB integrity.

### Mechanistic target of rapamycin (mTOR)

**Fig. 7:**
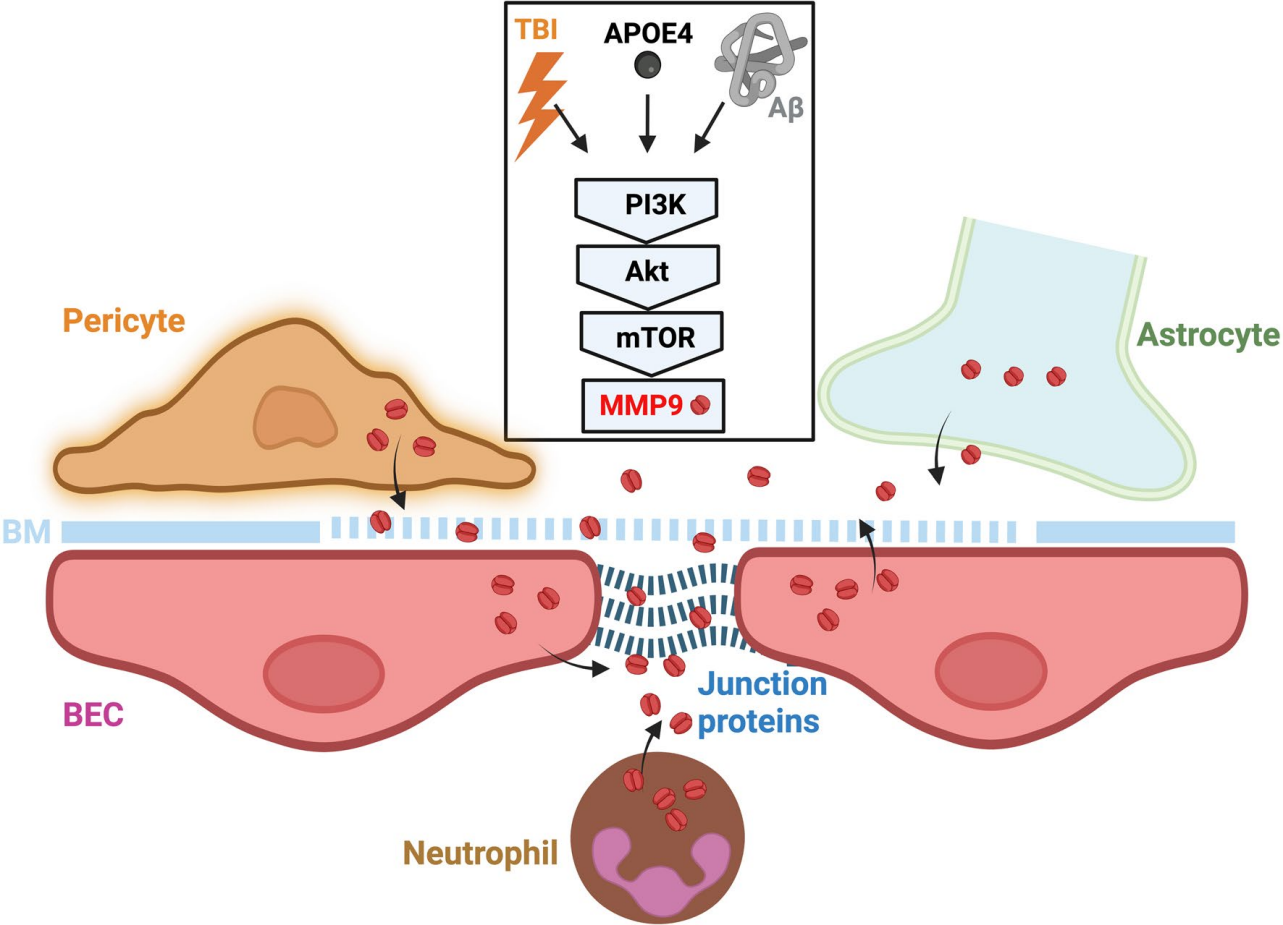
Upregulation of MMP-9 by mTOR signaling and proteolytic disruption of BBB. Following TBI and in AD, mTOR signaling pathway is activated, leading to upregulation of MMP-9, which disrupts the basement membrane (BM) and junction proteins. Neutrophils are a major source of MMP-9. MMP-9 can also be produced from BECs, astrocytes, and pericytes. Created with BioRender.com

mTOR is a highly conserved serine/threonine kinase that regulates many fundamental cell processes, from protein synthesis to autophagy. Deregulated mTOR signaling is implicated in the progression of cancer and diabetes [138] as well as the aging process [139]. In the CNS, mTOR plays a key role in nervous system development, and its activity is essential for normal brain function. However, mTOR hyperactivity can be detrimental to brain function [140]. Studies have demonstrated that mTOR activation is an early event in the development of AD and that increased mTOR signaling contributes to Aβ and tau aggregation, inflammation, and oxidative stress [141, 142]. Consequently, inhibiting mTOR facilitates the resolution of amyloid and tau aggregates and improves cognitive dysfunction [141, 142]. A detailed analysis of the findings points to mTOR as a critical mediator of cerebrovascular dysfunction [143]. mTOR drives cerebrovascular dysfunction, resulting in cerebral blood flow deficits and BBB breakdown; therefore, inhibition of mTOR activity protects BBB integrity [143]. In hAPP(J20) mice with AD and LDLR^−/−^ mice with vascular cognitive impairment, inhibition of mTOR with rapamycin abrogates BBB breakdown [144], restores neurovascular coupling and improves memory function [145]. The mechanism by which blocking the mTOR pathway protects BBB integrity involves increasing the expression of tight junction proteins and decreasing the activity of MMP-9 [144] (Fig. 7). mTOR inhibition also enhances LRP1 expression and Aβ efflux, thereby ameliorating Aβ accumulation in the brain [146]. mTOR may mediate the effects of *APOE4* on the BBB, as mTOR inhibition reduces proinflammatory pathways in brain vasculature and restores BBB integrity in the *APOE4* transgenic mice [147]. Like what is observed in AD, mTOR is activated after mTBI [148], and activation of mTOR complex 1 (mTORC1) impairs cognitive performance following mTBI [149]. These findings suggest a detrimental role for mTOR in the neurobehavioral performance following TBI and indicate that mTOR could be a potential therapeutic target. Indeed, pharmacological inhibition of the mTOR pathway in animal models of TBI has been shown to be beneficial for ameliorating TBI-associated symptoms and inflammatory responses [150]; however, whether this beneficial effect extends to preserving BBB function remains unknown.

The beneficial effects of mTOR inhibition in TBI, AD, and aging models are mainly observed with systemic inhibition. The specific cell types responsible for these effects remain unclear. mTOR signaling is activated in endothelial cells in aged mice. Importantly, endothelial cell‐specific deletion of mTOR reverses age‐related arterial dysfunction and MMP-9 expression (151), but also induces aging-associated phenotypes in the hematopoietic system [152]. These findings demonstrate that mTOR signaling in endothelial cells is crucial for maintaining the hematopoietic niche, while its hyperactivity harms endothelial function.

Systemic mTOR inhibition is linked to multiple side effects, some of which may result from mTOR inhibition in peripheral tissues [153]. Developing strategies to selectively inhibit mTOR in the brain while sparing its activity in other tissues could help mitigate the systemic effects of mTOR inhibitors [154]. In the brain, mTOR is expressed and activated in various cell types, including neurons [155], and its inhibition may affect their function, potentially contributing to side effects and toxicity. For example, while Rapamycin pretreatment prevents BBB disruption after cerebral ischemia, it also substantially increases the size of the infarcted cortical area [156], highlighting the critical role of mTOR in neuronal survival and the need for BEC-specific targeting.

**Fig. 8:**
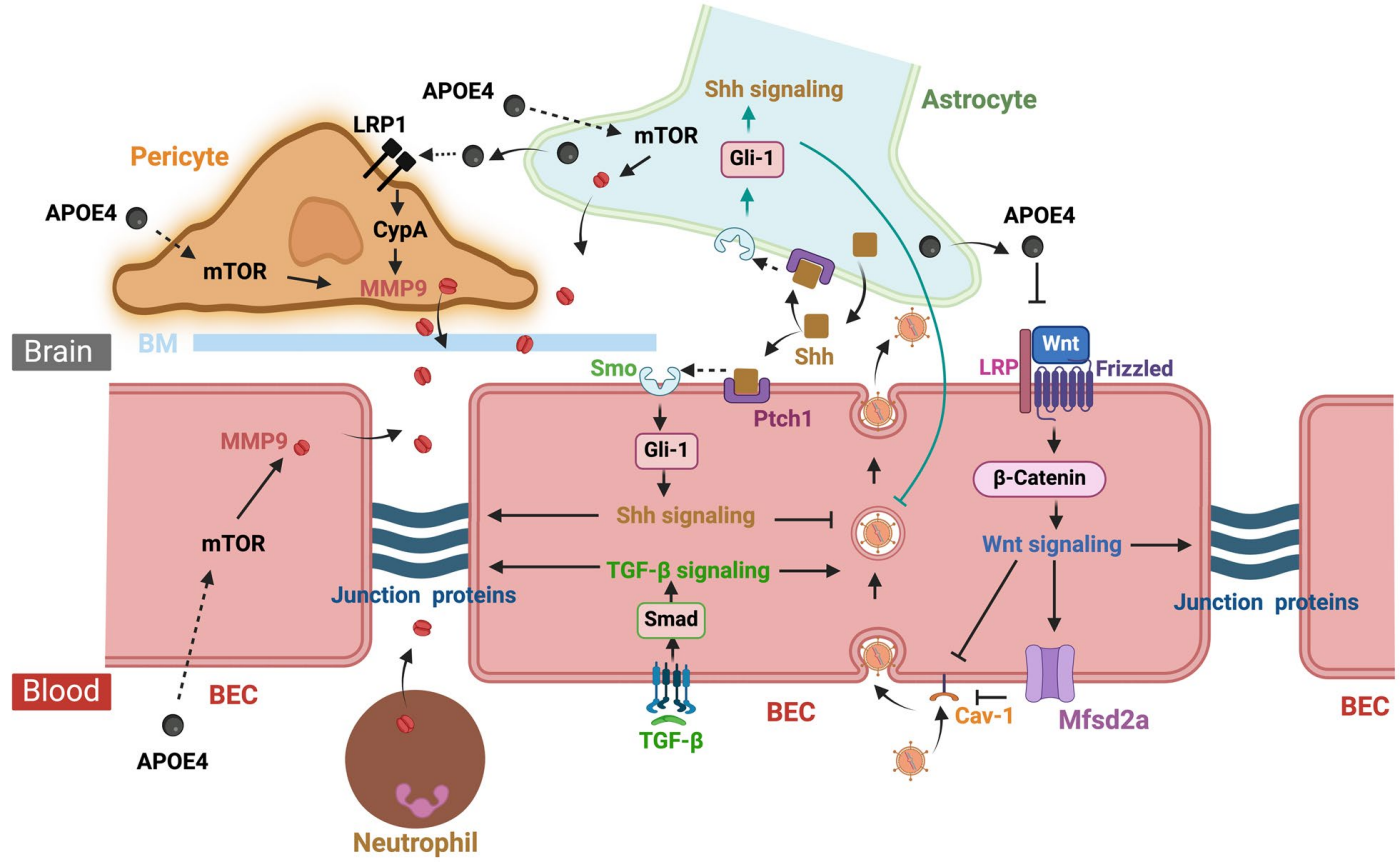
Major common pathways regulating BBB integrity after TBI and in AD. The BBB relies on three key structures for its physical barrier function: the BECs, tight junctions between BECs, and the basement membrane. The BECs exhibit low levels of vesicle trafficking, limiting transcellular transport or transcytosis, while tight junctions prevent uncontrolled paracellular passage. The basement membrane connects BECs with pericytes and astrocytes, forming the neurovascular unit and facilitating communication within this network. The function and integrity of the BBB are governed by multiple interacting pathways. Dysregulation of these pathways following TBI or in AD leads to increased BBB permeability. APOE4 and mTOR signaling trigger the activation of MMP-9, which degrades junction and basement membrane proteins, promoting uncontrolled paracellular passage. In contrast, activation of TGF-β, Shh, or Wnt signaling upregulates the expression of junction proteins, enhancing BBB stability and reducing this passage. Mfsd2a, a lipid transporter, cell-autonomously suppresses transcytosis at the BBB. Loss of Mfsd2a expression increases transcellular transport, while promoting its expression—such as through overexpression or WNT signaling—reduces this transport. Created with BioRender.com

The molecular pathways underlying BBB disruption are highly complex and involve multiple signaling cascades that regulate different cell types and components of the neurovascular unit (Fig. 8). The disruption of the BBB is not solely a result of endothelial cell dysfunction but is influenced by intricate crosstalk between endothelial cells, pericytes and astrocytes. This dynamic interplay contributes to the progressive nature of BBB dysfunction, ultimately influencing the disease outcomes in TBI or AD.

## Repairing the BBB attenuates neurodegeneration

Given the critical role of the BBB in maintaining neural health, repairing and restoring its integrity represents a promising therapeutic strategy for neurological diseases with BBB dysfunction. Various approaches have been explored, including the use of gene therapy, pharmacological agents, and regenerative medicine techniques.

### Targeting key regulators of BBB function

Gene therapy approaches and pharmacological drugs targeting the key factors involved in BBB function discussed above can affect BBB integrity. These approaches, when improving BBB integrity and ameliorating BBB dysfunction, typically lead to attenuated pathology and functional improvement (Table 1).

**Table 1:** Common molecular pathways involved in BBB dysfunction in TBI and AD

Pathways	Regulation after TBI	Regulation in AD	Genetic manipulation		Pharmacological intervention	
			TBI	AD	TBI	AD
ApoE4			Expression impairs BBB repair [71]	Expression leads to BBB breakdown [70]		ApoE antisense oligonucleotides [81], siRNAs [82] or antibodies [83] reduce Aβ and tau pathology and rescue cerebrovascular dysfunction [83]
TGF-β	Increased [86]	Impaired [86]			Treatment with an inhibitor prevents BBB leakage and reduces neurological deficits [34]	
Mfsd2a	Reduced [96–98]	Decreased [99]	Overexpression improves BBB function, and abolishes neurologic impairment [96]	Overexpression reverses learning and memory impairments [102]		
Wnt	Increased [117]	Impaired [110]		Activation restores BBB function and ameliorates AD pathology [119]	Activation mitigates BBB disruption and improves outcomes [98]	
MMP-9	Increased [126]	Upregulated [72, 127]	Ablation decreases BBB permeability, and improves memory [126]	Deletion improves memory [128]		Treatment with an inhibitor improves memory [128]
Shh	Impaired [131]			Blocking reduces neuronal apoptosis and improves learning and memory [136]	Shh treatment attenuates BBB dysfunction and promotes neurological recovery [131]	
mTOR	Activated [148]		Activation worsens neurocognitive outcome [149]		Inhibition ameliorates symptoms and inflammation [150]	Inhibition abrogates BBB breakdown [144], and reverses memory deficits (145)

### Stem cell therapy

Regenerative medicine techniques, such as stem cell therapy, hold potential for regenerating damaged endothelial cells and restoring BBB integrity. Different types of stem cells have been tested, and their effects on the BBB are summarized below.

#### Endothelial progenitor cells (EPCs)

EPCs migrate and home to the area of injury and can further proliferate and differentiate into mature endothelial cells to promote angiogenesis/vasculogenesis [157]. EPCs contribute to endothelial maintenance by serving as a cellular reservoir for replacing dysfunctional endothelial cells or by releasing angiogenic growth factors [158]. The number of EPCs in the peripheral blood has been proposed as a possible surrogate marker of vascular function after TBI [159]. Controversial results have been reported regarding whether the number of EPCs in the peripheral blood is increased or decreased in AD patients [158]. Nevertheless, the protective effects of EPCs on BBB integrity are consistent in both TBI and AD models. Intracerebroventricular transplantation of EPCs upregulates tight junction proteins, reduces BBB leakage, and improves neurological outcomes after TBI [160]. Similarly, stereotaxic injection of EPCs into the hippocampus improves BBB tight junction function, stimulates angiogenesis, and promotes Aβ clearance, ultimately improving cognitive function in APP/PS1 transgenic mice [161]. Interestingly, infusion of EPC-derived exosomes recapitulates these beneficial effects [162], suggesting that exosomes may participate in the protective response of EPCs.

#### Mesenchymal stem cells (MSCs)

MSCs are heterogeneous, multipotent adult cells that can be isolated from bone marrow and are capable of directional differentiation into mesenchymal and nonmesenchymal tissues [163]. They promote the regeneration of damaged tissues by inhibiting inflammation, secreting trophic factors, and recruiting local progenitor cells to replace lost cells, thus playing an important role in tissue regeneration [163]. Transplantation (via intravenous injection) of MSCs inhibits TBI-induced BBB dysfunction through preservation of cerebral endothelial adherens junctions and tight junctions [164]. The protective effect is mediated via tissue inhibitor of matrix metalloproteinase-3 (TIMP3), which is released by transplanted MSCs. Intravenous administration of recombinant TIMP3 preserves BBB integrity and improves neurocognitive function in TBI animals [164, 165]. Conversely, knockdown of TIMP3 expression in MSCs blocks the beneficial effects of the MSCs [164]. Therefore, TIMP3 likely plays a critical role in protection against TBI.

#### Hematopoietic stem cells (HSCs)

HSCs are undifferentiated cells located within hematopoietic tissues that are responsible for generating blood and immune cells. Transplantation of HSCs and progenitor cells isolated from wild-type mice preserves BBB integrity, reduces Aβ plaques and neuroinflammation, and rescues memory and neurocognitive decline in 5 × FAD mice [166]. These effects are believed to be mediated by microglia and endothelial cells, which exhibit significant decreases in disease- and neurodegeneration-associated gene expression.

#### Neural stem cells (NSCs)

NSCs are the stem cells of the nervous system. They can self-renew and generate progeny that differentiate into neurons, astrocytes, and oligodendrocytes [167]. They contribute to the formation of the nervous system during development. In adulthood, their numbers decrease, and they become confined to specific brain regions, remaining primarily in a quiescent state [168]. Nevertheless, the discovery of the ability of NSCs to differentiate into neural cell types has opened new therapeutic avenues for replacing lost or damaged CNS cells, especially through NSC transplantation. For example, transplanting human NSCs via intranasal administration rescues pericyte loss and mitigates Aβ pathology in APP/PS1 transgenic mice [169]. The BBB-repairing effects of NSCs are well-documented in stroke models, where they have been shown to enhance neurovascular integrity and reduce BBB permeability [170]. NSCs can be differentiated directly from pluripotent stem cells, such as induced pluripotent stem cells (iPSCs) [171, 172] and embryonic stem cells [173, 174]. They have been shown to reduce pathology and improve neurological function in TBI and AD models.

In summary, repairing the BBB can be achieved through various approaches and generally proves beneficial in both TBI and AD. In TBI, restoring BBB integrity helps mitigate secondary injury processes, reduce inflammation, and promote neural recovery. Likewise, in AD, improving BBB function has been shown to decrease Aβ and tau accumulation, reduce neuroinflammation, and preserve cognitive function.

## Discussion

Our current understanding of TBI and AD is still incomplete, as evidenced by the failure of many recent clinical trials evaluating therapies for these conditions. Similarly, although the appreciation of the significance of BBB dysfunction has been increasing [30–32], the exact role of BBB dysfunction in the pathogenic cascades of these diseases remains elusive. The complexity of the interactions among various cellular, molecular, and environmental factors in TBI and AD pathogenesis adds to the challenge. These clinical trial failures underscore the complexity of these diseases and the need for deeper investigation into their underlying mechanisms. Therefore, a primary objective for future and ongoing research is to enhance our understanding of the pathophysiological pathways underlying TBI and AD and the role of BBB dysfunction [175]. This involves not only identifying key molecular players and pathways involved in BBB disruption, but also understanding how these factors interact with other pathological processes and contribute to disease progression.

Notably, although accumulating evidence suggests that targeting BBB dysregulation could be a therapeutic strategy, no drugs designed explicitly to target BBB dysfunction have yet undergone clinical trials [27]. This is due not only to a limited comprehension of the molecular mechanisms underlying BBB dysfunction but also to a lack of advanced research tools [176]. Therefore, incorporating recent advancements in emerging technologies in BBB research will facilitate the study of BBB dysfunction and associated pathologies. For example, RNA-seq analysis has aided in the discovery of novel BBB regulators during aging [177] and inflammation [178], although the role of these regulators in TBI and AD still needs to be further investigated.

Another factor contributing to the current lack of BBB-targeting therapies is the absence of appropriate models, particularly human-based ones, which also hinders progress in understanding TBI and AD pathology, as well as BBB dysfunction. Animal models of TBI and AD are essential for elucidating disease mechanisms and testing therapeutic strategies; however, recent clinical trial failures underscore the limitations of current models [179, 180]. In response, initiatives such as the Model Organism Development and Evaluation for Late-onset Alzheimer’s Disease (MODEL-AD) Consortium [181] are focused on developing new mouse models to better represent the complex and heterogeneous mechanisms of these diseases. For example, the APP knock-in (KI) mouse model, which expresses human Aβ in the endogenous mouse APP gene, offers a more physiologically relevant model of sporadic AD [182]. This model has been demonstrated to be a more robust platform for investigating the interactions between environmental risk factors, such as TBI, and genetic predispositions associated with APP [183].

Recent advancements in stem cell and tissue engineering technologies have facilitated the use of organoids to model human diseases [184]. Human iPSC-derived 3D brain organoids, developed from patient-derived cells, replicate key aspects of human brain cytoarchitecture and human-specific AD pathology that cannot be reproduced in animal models [185]. These organoids provide unique opportunities to study AD and to develop and test therapeutic strategies in a humanized model system [185, 186]. They also represent a promising alternative for modeling the BBB [187]. Furthermore, they hold potential as a novel platform for studying TBI, either through direct injury induction [188] or following xenotransplantation into animals [189]. These newly developed models complement traditional animal models and offer significant potential to facilitate a more precise and comprehensive understanding of both TBI and AD.

The BBB is highly heterogeneous, exhibiting varying sensitivity to insults across brain regions [190]. Recent evidence suggests that the hippocampus, which is one of the first brain regions affected in AD, is particularly vulnerable due to BBB weakness [191]. TBI-induced BBB disruption is most pronounced in the hippocampus and frontal cortex [192]. Given the critical role of the hippocampus in learning and memory, the weakness and vulnerability of the BBB in this region may contribute to the detrimental effects of insults, such as TBI, on hippocampus-dependent functions [191]. Future investigation into the mechanisms underlying this vulnerability could therefore enhance our understanding of BBB function.

The current understanding of neurological diseases stems primarily from studies examining maladaptive changes, leading to the development of therapeutics aimed at reversing these effects [193]. Recent research, however, has shifted focus to include not only the accumulation of damage over a lifespan but also brain resilience, i.e., the ability of the brain to accommodate a certain degree of damage before clinical symptoms emerge [194]. This new approach could pave the way for novel therapeutic interventions that target mechanisms that support resilience rather than attempting to restore lost brain functions or structures, which has often proven unsuccessful [194–196]. The brain vasculature and BBB are integral to brain resilience [194, 197]. The integrity of the BBB is crucial in determining stress vulnerability and resilience [198]; for example, it influences the resilience of female animals to stress [199] and neurological impairments after TBI [200]. These findings highlight the importance of BBB resilience in preventing chronic dysfunction. Further studies on BBB resilience may not only provide new therapeutic approaches to enhance brain protection against neurodegenerative disorders but also offer novel insights into BBB dysfunction in disease.

In summary, embracing new advancements in emerging technologies and incorporating new models designed to mimic aspects of AD and TBI could be leveraged to further explore the mechanisms of BBB dysfunction and the link between TBI and AD.

## Conclusion

TBI is a potent risk factor for AD, with BBB dysfunction potentially serving as the underlying mechanism linking TBI to AD. Several common molecular factors and pathways have been found to mediate BBB dysfunction in both TBI and AD. Repair of the BBB through targeting these key regulators and stem cell therapy generally proves beneficial under both conditions. As such, BBB dysfunction is considered a pivotal factor in the pathogenesis of both TBI and AD, serving as a promising therapeutic target and an underlying mechanism linking these two conditions. Interventions aimed at repairing the BBB could therefore offer significant therapeutic benefits, potentially slowing disease progression and improving outcomes for patients with TBI and/or AD.

## Data Availability

Not applicable.

## Abbreviations

Aβ: Amyloid-beta
AD: Alzheimer’s disease
APOE4: Apolipoprotein E4
APP: Amyloid precursor protein
BECs: Brain endothelial cells
CTE: Chronic traumatic encephalopathy
EPCs: Endothelial progenitor cells
HSCs: Hematopoietic stem cells
iPSCs: Induced pluripotent stem cells
Mfsd2a: Major facilitator superfamily domain containing 2A
MMP-9: Matrix metalloproteinase-9
MSCs: Mesenchymal stem cells
mTOR: Mechanistic target of rapamycin
NSCs: Neural stem cells
Shh: Sonic hedgehog
TBI: Traumatic brain injury
TGF-β: Transforming growth factor β
